# Gut–Heart Axis: Microbiome Involvement in Wild-Type Transthyretin Amyloidosis

**DOI:** 10.3390/ijms27093763

**Published:** 2026-04-23

**Authors:** Itzel Ivonn López-Tenorio, Luis Alejandro Constantino-Jonapa, Samuel Jaimez-Alvarado, Fernando Hernández-Quiroz, Esteban Jorge-Galarza, Alma Reyna Escalona-Montaño, Amedeo Amedei, Rodrigo Soria-García, Enrique Alexander Berrios-Barcenas, María Magdalena Aguirre-García

**Affiliations:** 1 Unidad de Investigación UNAM-INC, División de Investigación, Facultad de Medicina UNAM, Instituto Nacional de Cardiología Ignacio Chávez, Mexico City 14080, Mexico; itzeltenorio20@hotmail.com (I.I.L.-T.); biologia0712@gmail.com (L.A.C.-J.); samueljaal@gmail.com (S.J.-A.); fernando.hernandezquiroz1@gmail.com (F.H.-Q.); almaescalona@comunidad.unam.mx (A.R.E.-M.); 2 Department of Outpatients Care, Instituto Nacional de Cardiología Ignacio Chávez, Mexico City 14080, Mexico; esjoga@yahoo.com.mx (E.J.-G.); rosogar92@gmail.com (R.S.-G.); 3 Department of Experimental and Clinical Medicine, University of Florence, 50134 Florence, Italy; amedeo.amedei@unifi.it; 4 Network of Immunity in Infection, Malignancy and Autoimmunity (NIIMA), Universal Scientific Education and Research Network (USERN), 50139 Florence, Italy; 5 SBE-Hospital Español, Mexico City 11520, Mexico

**Keywords:** gut microbiome, cardiovascular disease, transthyretin amyloidosis, cytokines, lipopolysaccharide, cardiac amyloidosis, transthyretin, free fatty acids

## Abstract

Cardiac amyloidosis is a rare and progressive condition characterized by the extracellular deposition of amyloid fibrils in multiple organs. Wild-type transthyretin amyloidosis (ATTR-wt) is the most common type affecting subjects above 60 years old. Recent and growing evidence suggests a potential link between GM and cardiac amyloidosis. In this scenario, the aim of the present study is to characterize the gut microbiota (GM), related metabolites and inflammatory biomarkers in ATTR-wt patients. In the ATTR patients we identified *Prevotella_9* as the core OTUs (Operational Taxonomic Unit) of this group, alongside *Prevotella 7*, *Prevotellaceae_UCG-003* and *Prevotellaceae_NK3B31*. In addition, there were increased levels of long fatty acids, including tetradecanoic, hexadecanoic and octadecanoic acids, in the ATTR group. The data obtained suggest that ATTR patients have an altered gut microbiota that could be used as a potential biomarker in metabolic and cardiovascular diseases, as well as a potential predictor of adverse prognosis in ATTR patients. In addition, the intestinal dysbiosis in ATTR patients could be associated with low-grade endotoxemia promoting a pro-inflammatory state due to the translocation of bacterial components, such as LPS (lipopolysaccharide), into blood circulation.

## 1. Introduction

Cardiac amyloidosis is a rare and progressive condition characterized by the extracellular deposition of amyloid fibrils in multiple organs, including the kidney, liver, nervous system, and heart [1,2]. In the myocardium, these deposits disrupt tissue architecture and impair cardiac function, leading to increased chamber stiffness, conduction abnormalities, and progressive diastolic dysfunction. Cardiac amyloidosis can progress to heart failure with reduced ejection fraction (HFrEF) or can trigger fatal arrhythmias [3].

Two major types of cardiac amyloidosis have been identified: light chain amyloidosis (AL) and transthyretin amyloidosis (ATTR). AL is caused by the deposition of misfolded immunoglobulin light chains produced by dysfunctional plasma cells in the bone marrow [4]. On the contrary, the ATTR results from the misfolding and deposition of transthyretin (TTR), a transport protein primarily synthesized in the liver [5]. ATTR is further classified in wild-type (ATTR-wt) and hereditary (ATTR-h) forms [6]. ATTR-wt is associated with aging and represents the most common form in patients over 60 years of age. In contrast, ATTR-h is an autosomal dominant disease caused by pathogenic variants in the TTR gene and is often associated with younger patients [7].

The broad spectrum of clinical manifestations and the ability of cardiac amyloidosis to mimic more common cardiac conditions often lead to diagnostic delays and, consequently, deferred treatment. As a consequence, the prognosis is generally poor, with an estimated median survival of six months for AL and between four and ten years for ATTR-h and ATTR-wt [8].

ATTR-wt has a heterogeneous life prognosis shaped by age at diagnosis, disease stage and presence of heart failure (HF) at presentation; in the Healthcare Amyloidosis European Registry analysis, patients diagnosed at ≤65 years have better post-diagnosis survival than those diagnosed after 65 years, despite meaningful differences in diagnostic delay and baseline HF signs [9]. In the THAOS analysis, 30-month survival was numerically higher in patients treated with selective TTR stabilizing drug vs. untreated patients overall [10]. Finally, biopsy-proven series highlight that even when overall survival is better than AL, HF readmissions remain high in ATTR underscoring ongoing morbidity that influences “prognosis of life” beyond mortality alone [11].

Emerging evidence suggests a potential link between GM and cardiac amyloidosis. In experimental models, doxycycline treatment has been shown to decrease beta-amyloid deposition and modify GM composition [12,13]. Furthermore, the protease subtilisin derived from *Bacillus subtilis* has the ability to cleave the ATTR protein, generating amyloidogenic fragments [14].

The gut microbiome (GM) comprises a highly diverse community of microorganisms that live in symbiosis with the host and contribute to its health by fermenting insoluble dietary fiber, synthesizing essential vitamins, modulating immune response, and maintaining intestinal barrier integrity and homeostasis [15]. On the contrary, the dysbiosis, an imbalance in the composition or function of the GM, can increase intestinal permeability, promote systemic inflammation and alter lipid metabolism [16,17,18].

In a 16S rRNA study comparing ATTR patients with and without cardiac involvement to controls, some differences were observed in the abundance of *Streptococcus*, *Lachnospiraceae*, and *Sellimonas*, while the controls showed higher *Methanosphaera*; notably, *Streptococcus* correlated with higher troponin, and *Lachnospiraceae* with lower BNP, supporting associations between specific taxa and markers of myocardial injury [19]. Using shotgun metagenomics plus untargeted serum metabolomics in ATTR-h, another study reported marked reductions in GABA and taurine, which are most pronounced among patients with cardiac amyloidosis, alongside decreases in commensals such as *Bifidobacterium pseudocatenulatum* and *Lactobacillus rogosae* and pathway signals implicating disrupted glutamate/taurine metabolism, supporting a GM–metabolite link to cardiac phenotypes [20].

Systemic inflammation associated with endotoxemia may promote TTR misfolding by generating an environment that increases both the formation and persistence of amyloidogenic species. First, lipopolysaccharide (LPS) can increase intestinal permeability through TLR4/CD14-dependent mechanisms, facilitating the translocation of microbial products into circulation [21]. Second, the amyloidogenic ability of nattokinase, a fibrinolytic subtilisin-like serine protease from *Bacillus subtilis natto* has been documented [22]. Third, inflammation increases oxidative stress, and experimental evidence shows that oxidative TTR modifications are associated with a greater propensity for aggregation and cytotoxicity in cardiomyocytes, supporting the idea that an inflammatory microenvironment can destabilize TTR and promote its misfolding [23]. Finally, the progression of deposition depends on clearance: macrophages/monocytes can endocytose and degrade it [24]. However, much of this evidence has been generated primarily in the context of ATTR with polyneuropathy.

In a pilot metabolomic study of hereditary ATTR-h, an altered free fatty acids (FFAs) profile was identified, with palmitic acid, a long-chain fatty acid (LCFA) significantly lower in patients compared to healthy controls, supporting the idea that the disease is associated with quantifiable changes in circulating FFAs [25]. Mechanistically, it has been shown that ω-3 and ω-6, also LCFAs, decrease the rate of TTR aggregation and generate fibrils with lower cytotoxicity than those formed without FFAs, suggesting that the “fatty acid environment” may influence amyloid properties [26]. Available evidence has focused primarily on LCFAs rather than short-chain fatty acids (SCFAs), which are considered the metabolites most closely linked to GM activity. Finally, because ATTR-wt commonly manifests as a HF, circulating FFAs independently predict adverse outcomes in large HF cohorts [27], so we included FFAs to better capture the systemic metabolic stress that could be related to ATTR-wt.

Despite these findings, the microbial involvement in cardiac amyloidosis pathophysiology remains poorly understood and the role of GM, derived metabolites, and inflammatory profile in patients with ATTR-wt has not been explored to date.

So, the aim of the present study was to characterize GM richness and diversity, profile key microbial metabolites like free fatty acids (FFAs), and assess systemic inflammation in patients with diagnosed ATTR-wt.

## 2. Results

### 2.1. Enrolled Participants

In this study 23 subjects were recruited and were divided into three study groups: eight ATTR patients, (two females and six males); seven patients with HFrEF (three F and four M); and eight healthy controls (two F and six M).

The ATTR group had an average BMI of 26.4 kg/m^2^, the HFrEF group had an average BMI of 24.5 kg/m^2^, and the control group had an average BMI of 22.68 kg/m^2^. Serum glucose levels were reported to be 91 mg/dL for ATTR patients, 93 mg/dL for HFrEF patients, and 94.28 mg/dL for the control group. The average total cholesterol level analyzed for the three groups was 155 mg/dL for ATTR patients, 131 mg/dL for HFrEF patients, and 208 mg/dL for the control group.

Regarding LDL cholesterol levels in the ATTR, HFrEF, and healthy groups, the values were 89 mg/dL, 74.4 mg/dL, and 137 mg/dL, respectively. HDL cholesterol levels were reported as 39.8 mg/dL for ATTR, 74.4 mg/dL for HFrEF, and 50.6 mg/dL for the healthy group. C-reactive protein levels were 7.3 mg/L in the ATTR group, 1.91 mg/L in the HFrEF group, and 0.51 mg/L in the healthy group. The average white blood cell count was 7 × 10^9^/L for ATTR patients, 5.9 × 10^9^/L for HFrEF patients, and 4.57 × 10^9^/L for the control group. The albumin levels in the ATTR, HFrEF, and healthy patients were 3.9 g/dL, 4.5 g/dL, and 4.47 g/dL, respectively. Regarding myocardial damage markers, the average troponin T level was 48.8 ng/L in the ATTR patients, 26 ng/L in the HFrEF patients, and 10.8 ng/L in the control group. NT-proBNP levels showed an average of 4344 pg/mL, 709 pg/mL and finally 8.9 pg/mL, respectively.

Two ATTR patients were obese and non-current smokers were recruited, however, three patients had a history of smoking and three were alcohol consumers. Within this same group, five patients had a diagnosis of dyslipidemia, six had systemic arterial hypertension (SAH), and five had type 2 diabetes mellitus (T2DM). None of the ATTR patients suffered a myocardial infarction (AMI), and only one patient reported a decrease in left ventricular ejection fraction.

In the HFrEF group, none of the patients showed obesity, two were smokers, and only one patient reported a history of alcohol consumption. Six patients had been diagnosed with dyslipidemia, and all of them had hypertension. Seven had a T2DM diagnosis, four had a history of myocardial infarction, and all presented with a reduced left ventricular ejection fraction (HFEF).

In the control group, none of the patients had any of the previously mentioned comorbidities.

The descriptive characteristics of the study subjects, as well as the variables analyzed, are summarized in Table 1.

### 2.2. Fecal Microbiota Characterization

**Figure 1:**
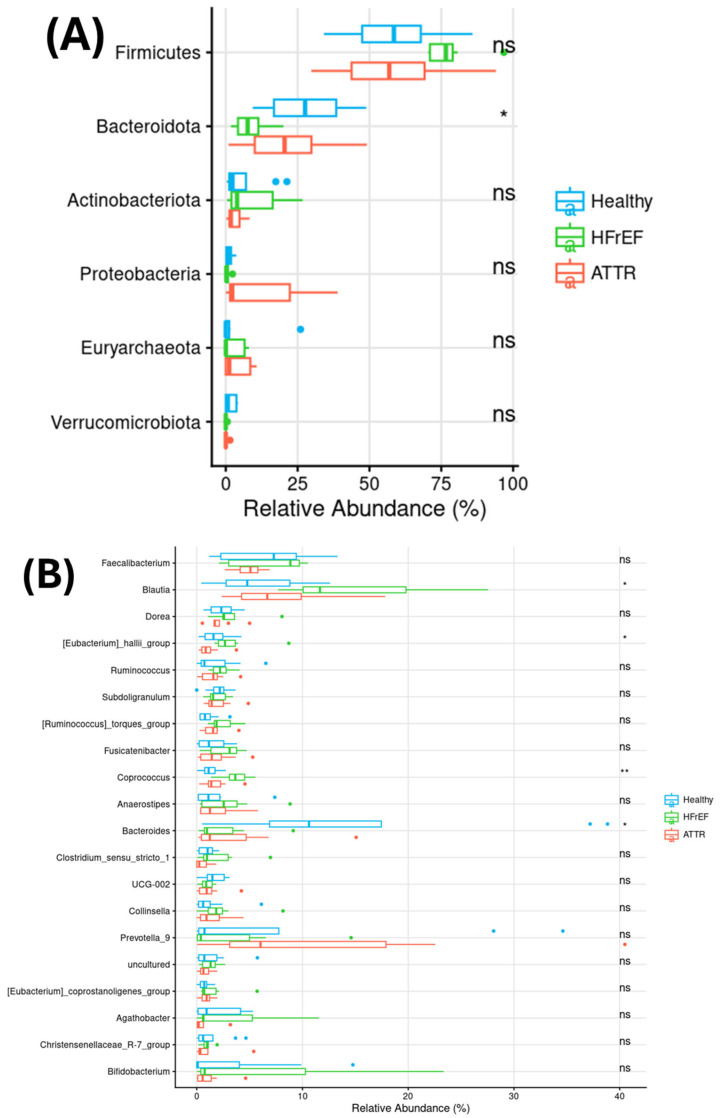
Relative abundance of the six most represented phyla in the GM (**A**) and of the twenty most abundant genera in the GM (**B**). Group 1: healthy “blue”, group 2: HFrEF “green” and group 3: ATTR “red”. The Kruskal–Wallis test was applied: * (*p* < 0.05), ** (*p* < 0.01).

The most predominant phyla, in all the enrolled individuals, correspond to *Firmicutes*, *Bacteroides*, *Bacteroidota*, *Actinobacteria*, *Proteobacteria*, *Euryarchaeota* and *Verrucromicrobia*. There is a *bacteroidota* decrease in HFrEF patients in comparison with ATTR and healthy groups. However, at the genus level, the more predomominant genera are *Faecalibacterium*, *Blautia*, *Dorea*, [*Eubacterium*]_*hallii*_group, *Ruminococcus*, *Subdoligranulum*, [*Ruminococcus*]_*torques*_group, *Fusicatenibacter*, *Coprococcus*, *Anaerostipes*, *Bacteroides*, *Clostridium*_*sensu*_*stricto*_*1*, *UCG-002*, *Collinsella*, *Prevotella_9*, *uncultured*, [*Eubacterium*]_*coprostanoligenes*_group, *Agathobacter*, *Christensenellaceae_R-7*_group and *Bifidobacterium* (Figure 1A).

A significant increase in the abundance of the *genera Blautia*, [*Eubacterium*]*_hallii*_group, and *Coprococcus* was observed in the HFrEF group compared with both the ATTR group and HCs. On the other hand, the *Bacteroides* genus decreased significantly in the HFrEF group followed by the ATTR group when compared to HCs (Figure 1B).

**Figure 2:**
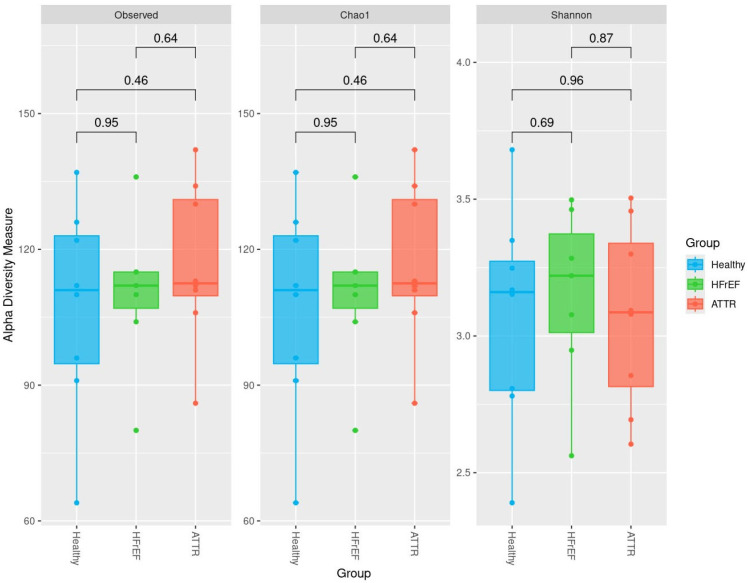
Analysis of alpha diversity of gut microbiota. Box plots showing alpha diversity indices in both GM between groups. Group 1: healthy “blue”, group 2: HFrEF “green” and group 3: ATTR “red”. The Wilcoxon rank-sum test was applied.

Alpha diversity analysis was performed using the observed OTU index, the Chao 1 index, and the Shannon index for the GM. We did not document significant differences regarding alpha diversity indexes (Figure 2).

**Figure 3:**
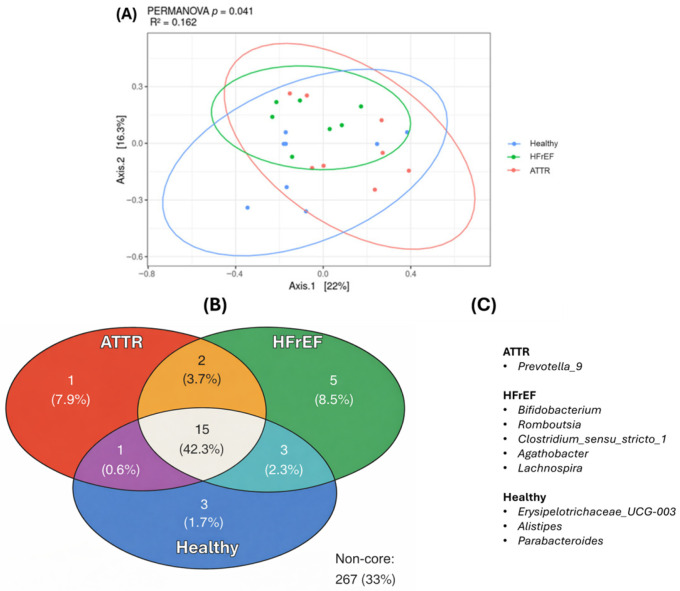
Principal coordinate analysis of beta diversity using the unweighted UniFrac index and a PERMANOVA test was applied (**A**). The Venn diagram (**B**) shows the core OTUs shared between the groups and (**C**) shows the unique core OTUs of each group.

Beta diversity analysis was performed using principal coordinates (PCoA) and the unweighted Unifrac dissimilarity index along with the PERMANOVA test. A significant difference (*p* = 0.041) was found in the GM of the study groups for all three groups, showing that the R-squared value was 0.162, (Figure 3A).

Using the ampvis2 package, 2.0 we determined the core OTUS of each group. It was observed that ATTR patients and HCs share one genus, patients in the HFrEF group share three genera with the HC group, the ATRR group and the HFrEF group share two genera. Finally, the three groups share a total of 15 genera.

It was found that certain genera were specific to each group: (*Prevotella_9*) in the ATTR group; *Bifidobacterium*, *Romboutsia*, *Clostridium sensu stricto_1*, *Agathobacter*, and *Lachnospira* in the HFrEF group; and *Erysipelotrichaceae_UCG-003*, *Alistipes*, and *Parabacteroides* present in the HC group (Figure 3B).

**Figure 4:**
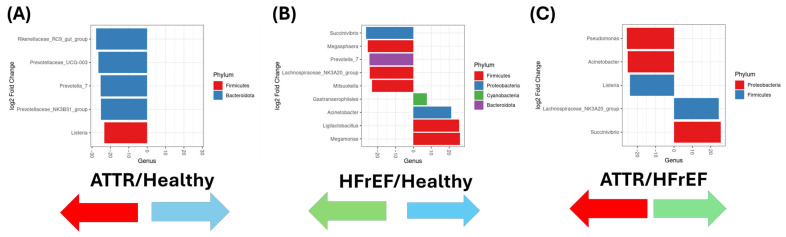
Differential abundance analysis of gut microbiome using DESEQ2 analysis. In both figures, negative fold change is associated with the first group of the comparison. while positive fold change is associated with the second group of the comparison. (**A**) Group 1: healthy “blue” versus group 3: ATTR “red”; (**B**) group 2: HFrEF “green” versus group 1: healthy “blue”; and (**C**) group 3: ATTR “red” versus group 2: HFrEF “green”.

In addition, DESEq2 was used to analyze differential genera present in GM from the study groups. Five distinct genera were identified between the ATTR group and the healthy group (in detail: *Rikenellaceae_RC9_gut*_group, *Prevotellaceae_UCG-003*, *Prevotella_7*, *Prevotellaceae_NK3B31*_group, and *Listeria*), of which three were from the phylum *Bacteroidota* and one was from the phylum *Firmicutes*, respectively (Figure 4A). For the HFrEF group there were five genera (*Succinivibrio*, *Megasphera*, *Prevotella_7*, *Lachnospiraceae NK3A20*_group, *Mitsuokella*) which belong to *Proteobacteria*, *Firmicutes* and *Bacteroidota*; *Prevotella_7* differed from the HC group since this group showed four *genera* (*Gastranaerophilales*, *Acinetobacter*, *Ligilactobacillus* and *Megamonas*) belonging to the *phyla Cyanobacteria*, *Proteobacter* and *Firmicutes*, respectively (Figure 4B).

Finally, when comparing the group of ATRR patients, they showed three genera (*Pseudomonas*, *Acinetobacter* and *Listeria*) which differed from the group of HFrEF patients (*Lachnospiraceae_NK3a20*_group and *Succinivibrio*), belonging to the phyla *Proteobacteria* and *Firmicutes* (Figure 4C). A comparison between ATTR patients with and without risk factors (hypertension, type 2 diabetes, and dyslipidemia) is shown in Supplementary Figures S1–S3. No significant differences were observed; however, *Ruminococcus_torques*_group and *Eubacterium_hallii*_group were found to be increased in ATTR patients with type 2 diabetes. The complete relative abundance of gut microbiota for each individual is provided in the Supplementary Table S1.

### 2.3. Circulating FFA Profile

A decrease in the concentration of short-chain fatty acids (SCFAs), acetic, isobutyric, valeric, propionic, isohexanoic, and butyric acids, was observed in the ATTR and HFrEF groups compared to the HC group. Furthermore, acetic acid levels decreased significantly (*p* = 0.024) in the HFrEF patients compared to the ATTR group. Conversely, isovaleric acid levels increased significantly (*p* = 0.044) in the ATTR and HFrEF groups compared to the healthy controls.

HFrEF patients showed lower concentrations of octanoic acid compared to the ATTR and HC groups. Conversely, no concentration of dodecanoic acid was observed in the ATTR and HC groups compared to the HFrEF group. The concentration of dodecanoic acid was decreased in the HFrEF group compared to the ATTR group and significantly lower (*p* = 0.027) compared to the HCs.

Long-chain fatty acids (LCFAs) such as tetradecanoic acid showed significantly increased concentrations (*p* = 0.013 and *p* = 0.019) in ATTR patients compared to the HFrEF and HC groups, respectively.

**Figure 5:**
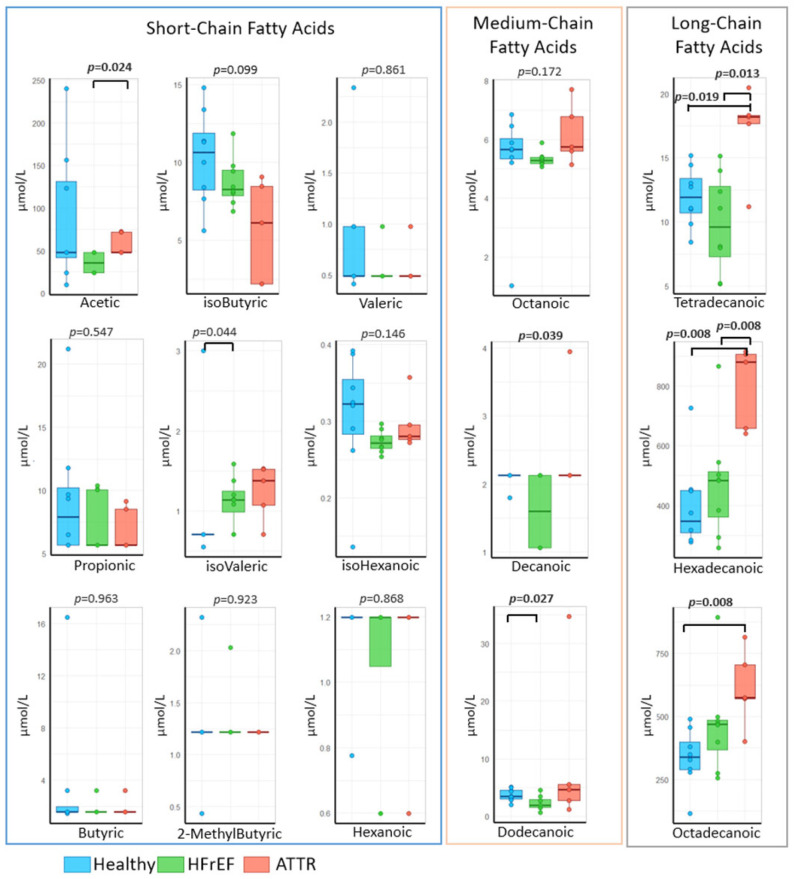
Concentration of short-, medium- and long-chain fatty acids in patients with group 1: healthy “blue”, group 2: HFrEF “green” and group 3: ATTR “red”. *p*-values were calculated using the Wilcoxon test for pairwise comparisons and the Kruskal–Wallis test for comparisons among multiple groups.

Lastly, the hexadecanoic acid also showed an increase in the ATTR group compared to the HFrEF and HC groups (*p* = 0.008). Of note, the concentration of octadecanoic acid also increased significantly in ATTR patients (*p* = 0.008) compared to the HCs (Figure 5).

### 2.4. Cytokine Signature Across the Study Groups

**Figure 6:**
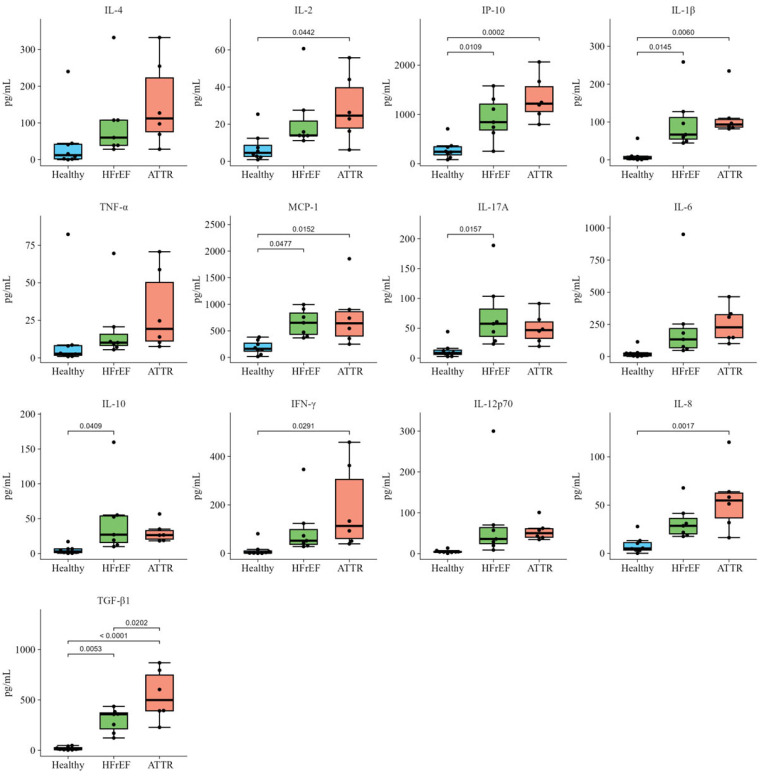
Cytokine levels of ATTR, HFrEF and healthy groups. The *p*-value was calculated using the Wilcoxon test.

Comparing the ATTR patients with the HFrEF and HC groups, we documented significant increased levels of different cytokines and chemokines, including IL-4 (112.25 pg/mL), IL-2 pg/mL (24.62 pg/mL), IL-1β (93.33 pg/mL), TNF-α (19.30 pg/mL), IL-17A (46.91 pg/mL), IL-6 (1226.83 pg/mL), IL-10 (26.44 pg/mL), IFN-γ (113.00 pg/mL), IL-12p70 (50.07 pg/mL), IL-8 (56.12 pg/mL), and TGF-β1 (498.70 pg/mL), and the chemokines IP-10 (1331.34 pg/mL) and MCP-1 (774.36 pg/mL). In addition, the cytokines IL-4 (60.09 pg/mL), IL-2 pg/mL (13.96 pg/mL), IL-1β (66.73 pg/mL), TNF-α (10.14 pg/mL), IL-17A (57.60 pg/mL), IL-6 (132.90 pg/mL), IL-10 (27.05 pg/mL), IFN-γ (52.18 pg/mL), IL-12p70 (35.91 pg/mL), IL-8 (32.39 pg/mL), and TGF-β1 (357.52 pg/mL) as well as the chemokines IP-10 (923.99 pg/mL) and MCP-1 (650.13 pg/mL) of the HFrEF patients showed significant increases when compared to the HCs, which showed values within normal ranges: IL-4 (12.25 pg/mL), IL-2 (24.62 pg/mL), IL-1β (4.53 pg/mL), TNF-α (2.77 pg/mL), IL-17A (8.79 pg/mL), IL-6 (17.15 pg/mL), IL-10 (3.02 pg/mL), IFN-γ (2.43 pg/mL), IL-12p70 (4.14 pg/mL), IL-8 (8.51 pg/mL), and TGF-β1 (11.07 pg/mL). The corresponding statistical data are shown in Figure 6.

### 2.5. Increased LPS Level in Patients with ATTR and HFrEF

**Figure 7:**
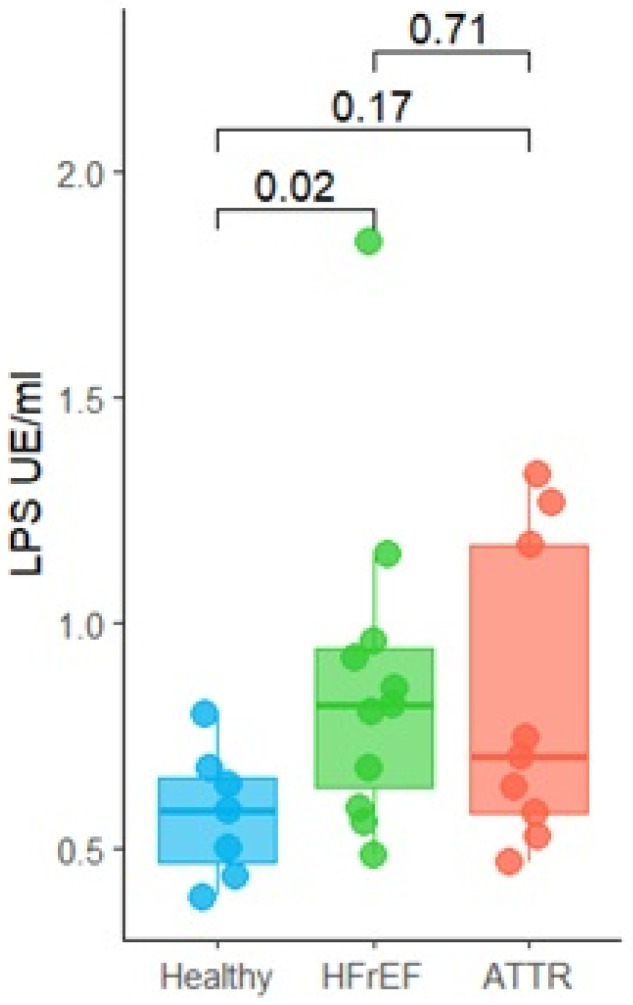
Determination of lipopolysaccharide (LPS) concentration, in units of (EU/mL), in serum of patients with ATTR cardiac amyloidosis and patients with reduced left ventricular ejection fraction HFrEF versus healthy subjects. Group 1: healthy “blue”, group 2: HFrEF “green” and group 3: ATTR “red”. Values are expressed as mean ± standard error. *p*-values were calculated using Wilcoxon tests; significant differences were defined as *p* ≤ 0.05.

Due to both ATTR and HFrEF groups having higher levels of serum cytokines, we measured the LPS levels in the serum. A significant increase in LPS concentration was observed in the serum of patients in the HFrEF group (*p* = 0.02) in comparison with the healthy group as shown in Figure 7. Although not significant, ATTR patients also had higher levels of LPS (*p* = 0.17).

### 2.6. Association Between Microbial Taxa, Fatty Acids and Cytokines

**Figure 8:**
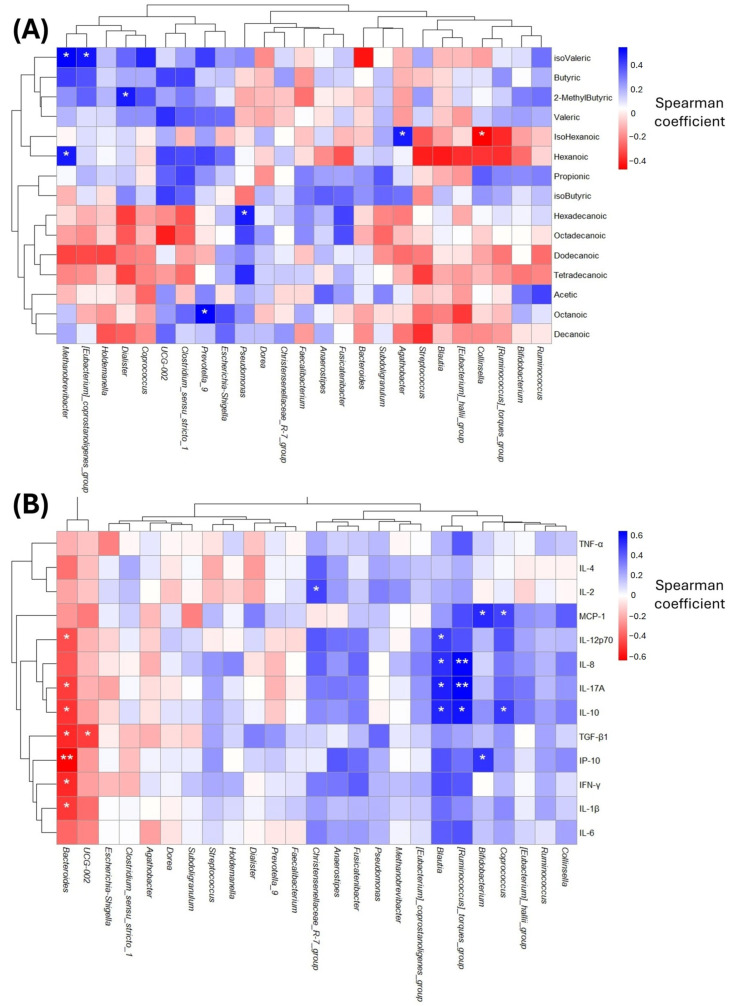
Spearman correlations among serum FFAs abundance and both GM (**A**) compositions. Correlations among serum cytokines levels and both GM (**B**). Spearman correlation was calculated between cytokines and the more abundant genus of gut microbiota. * (*p* < 0.05), ** (*p* < 0.01).

To analyze the relationship between gut microbiota and FFAs, a spearmen correlation heatmap was performed. A negative correlation between the *genus Collinsella* and isohexanoic acid was found. Additionally, some positive correlations were found, such as between hexanoic acid and isovaleric acid and the genus *Methanobrevibacter*. In addition, *Eubacterium* positively correlated with isovaleric acid, *Dialister* with 2-methylbutyric acid, *Prevotella_9* with octanoic acid, *Pseudomonas* with hexadecanoic acid, and *Agathobacter* with isohexanoic acid (Figure 8A).

Subsequently, we correlated the serum levels of anti-inflammatory and pro-inflammatory cytokines with the gut microbiota. A negative correlation was found between IL-1β, IFN-γ, TGF-β1, IL-10, IL-17A, IL-12p10, and IP-10 with *Bacteroides*, and a stronger association with *IP-10*. *UCG-002* was negatively associated with TGF-β1. On the other hand, several positive correlations were observed, including between *Christensenellaceae _R-7*_group and *IL-2*, *Blautia* and IL-10, IL-17A, IL-1L-8, and IL-12p10, and *Ruminococcus* and IL-1L-10, IL-17A, IL-8, and IL-12p10. In addition, *Bifidobacterium* was associated with IP-10 and MCP-1. Finally, *Coproccoccus* showed a positive association with IP-10 (Figure 8B).

### 2.7. Dysregulation of Key Enzymes and Metabolic Pathways in HFrEF and ATTR Patients

The functional enzyme predictions obtained from PICRUSt2 were analyzed using RStudio (version 4.4.1), according to the described methodology, to determine statistically significant differences using Welch’s *t*-test, using the healthy group to compare against HFrEF and ATTR independently. The comparisons against the HFrEF group showed four enzymes were significantly enriched: Fructuronate reductase (*p* = 0.006), Glyceroldehydrogenase (*p* = 0.0035), Liditol-2-dehydrogenase (*p* = 0.0319), and Shikimatedehydrogenase (*p* = 0.0075); in turn, in the HC group there were five significantly enriched enzymes: GDPL-fucose synthase (*p* = 0.0125), Malate dehydrogenase (*p* = 0.0099), Malatedehydrogenase (NADP) (*p* = 0.0038), X4 hydroxythreonine 4-phosphatedehydrogenase (*p* = 0.0068), and X4 phosphoerythronate dehydrogenase (*p* = 0.0162); only the UDP glucose 6-dehydrogenase enzyme showed enrichment in the healthy controls when compared to the HFrEF (*p* = 0.0094) and ATTR (*p* = 0.0394) groups (Supplementary Figure S1, Table 2).

Additionally, we calculated the z-scores of the same 20 enzymes to create a heatmap including row- and column-activated clustering to perform a general screening; firstly, the healthy and ATTR groups showed a direct clustering with respect to the HFrEF group, where the enzymes show three profiles, one for each group, whereas in the healthy group there are four enzymes: up-regulated UDP glucose 6-dehydrogenase, GDP L-fucose synthase, Malate dehydrogenase, and X4 phosphoerythronate dehydrogenase; n the HFrEF group there are four: Glycerol dehydrogenase, Fructuronate reductase, L iditol 2 dehydrogenase, and y Shikimate dehydrogenase. Finally, the ATTR group has ten enriched enzymes: S hydroxymethyl mycothiol dehydrogenase, Quinoprotein glucose dehydrogenase PQQ quinone, Gluconate 2 dehydrogenase, X3 hydroxyacyl CoA dehydrogenase, UDP glucuronic acid oxidase UDP 4 keto hexauronic acid decarboxylating, Glyoxylate reductase, X3 hydroxybutyrate dehydrogenase, S hydroxymethyl glutathione dehydrogenase, D malate dehydrogenase decarboxylating, and Tartrate dehydrogenase (Supplementary Figure S4).

Complementing the results obtained on enzyme enrichment, the metabolic pathways obtained from PICRUSt2 were analyzed, performing the same analyses used with the enzyme data; the HC group showed four significantly enriched pathways: biotin biosynthesis I (*p* = 0.0202), colanic acid building blocks biosynthesis (*p* = 0.0036), pyruvate fermentation to butanoate (*p* = 0.0246), and the superpathway of fatty acid biosynthesis initiation (*p* = 0.0153);the HFrEF group showed eight statistically significant routes: the Calvin–Benson–Bassham cycle (*p* = 0.0067), L-lysine biosynthesis I (*p* = 0.0034), chorismate biosynthesis I (*p* = 0.0034), coenzyme A biosynthesis I (*p* = 0.0066), fucose degradation (*p* = 0.0137), glycogen biosynthesis I (*p* = 0.0198), the superpathway of aromatic amino acid biosynthesis (*p* = 0.0035), and the superpathway of branched amino acid biosynthesis (*p* = 0.0167). One showed a tendency toward enrichment: glycogen degradation I (bacterial) (*p* = 0.0512). Only gluconeogenesis I showed enrichment in the HC group when compared to the HFrEF (*p* = 0.0162) and ATTR (*p* = 0.0340) groups when independently compared (Table 2).

The z-scores of the same 20 metabolic pathways were also calculated to create a heatmap, including row- and column-activated clustering for general screening. Firstly, the HC and ATTR groups showed a direct clustering with respect to the HFrEF group, where the metabolic pathways showed three profiles, one for each group, whereas the healthy group showed two pathways: up-regulated cholanyl acid building blocks biosynthesis and gluconeogenesis I; the HFrEF group showed ten pathways: fucose degradation, L-arginine biosynthesis II (acetyl cycle), L-lysine biosynthesis I, chorismate biosynthesis I, the superpathway of aromatic amino acid biosynthesis, the Calvin-Benson-Bassham cycle, glycogen biosynthesis I (from ADP-D-Glucose), glycogen degradation I (bacterial), the superpathway of branched amino acid biosynthesis, and coenzyme A biosynthesis I; and the ATTR group showed five routes; pyruvate fermentation to butanoate, L-arginine degradation II (AST pathway), fatty acid beta-oxidation I, the superpathway of glycolysis, and y heme biosynthesis I (aerobic) (Supplementary Figure S5).

## 3. Discussion

The causes of wild-type ATTR are still unknown, but the misfolding of proteins and their subsequent deposition by the immune system are key pathological characteristics. There are some reports that can induce the unfolding of certain proteins. Several factors can induce unfolding, like oxidative stress, infections and mutation in specific genes [28]. Recent evidence describes changes in the GM profile of patients with cardiovascular diseases, including heart failure, HFrEF, and ATTR, in the onset and progression of these pathologies. It has been observed that *Escherichia*/*Shigella* is increased in patients with cardiovascular diseases, indicating a GM dysbiosis [29]. We have previously documented that *Escherichia-Shigella* is increased in STEMI patients compared with healthy controls [30].

Our results documented no significant differences in alpha diversity indexes (OTUs observed, Chao1 and Shannon), but when analyzing beta diversity, a significant shift in ATTR patients compared with the healthy group was revealed, indicating that ATTR patients have an altered microbiota.

To analyze the gut microbiome of ATTR patients, we included two control groups: a healthy cohort and a cohort of patients with heart failure with HFrEF, since ATTR patients usually show reduced ejection fraction; however, despite sharing a similar clinical phenotype and pharmacological treatment, both groups differ in etiology.

We documented a significant decrease in the abundance of the phylum *Bacteroidota* in HFrEF patients. In this phylum there are both beneficial bacteria like *Bacteroides fragilis* bacteria, as well as harmful bacteria like *Porphyromonas gingivalis.* At the genus level, there is an increase in the genera *Blautia*, *Eubacterium* and *Coprococcus* in HFrEF patients. Although *Blautia* species can metabolize dietary fiber into SCFAs, like *B. luti* and *B. wexlerae*, *Blautia* has been associated with patients with irritable bowel disease [31], as well with obesity, diabetes and cancer. *Eubacterium* also is a SCFA producer; however, it has been related with an important function to maintain an intestinal metabolic balance, mainly through the production of butyrate. Similarly, *Coproccocus* is another key bacteria that is decreased in patients with inflammatory bowel disease and non-alcoholic fatty liver disease, along with “healthy” bacteria such as *Lactococcus* and *Ruminococcus* [32].

Bacteroides were found to be reduced in both ATTR and HFrEF patients. This genus has a broad variety of both bacteria and metabolic functions. *B. fragilis* has been associated with the activation of microglia and symptoms of Alzheimer’s disease in murine models [33]. On the other hand, other *Bacteroides* species have a relevant role in maintaining good intestinal health. Non-pathogenic *B. fragilis* strains have the capability to diminish both systemic and local inflammation, enhancing intestinal permeability by increasing tight junction proteins [34]. These data indicate that *Bacteroides* species could be associated with good health, especially in Mexican people, as we have previously documented [30]. Taken together, these findings indicate that *Bacteroides* spp. presenting in healthy Mexican individuals could represent beneficial GM members.

Analyzing the core OTUs of each group, we found that *Prevotella 9* is exclusive to ATTR patients. Originally, the genus *Prevotella* included a wide variety of members, but in recent years this genus has been split in several clades [35]. *Prevotella_9* (or Segatella in a recent database) are mainly intestinal bacteria, with *Prevotella copri* (or *Segatella copri)* being the most representative member. This species has a contrasting role due to being associated with diseases like rheumatoid arthritis and Parkinson’s disease [36]. Here, *Prevotella_9* was detected only in ATTR patients but not in HFrEF patients, likely mirroring the different etiologies. Alongside *Prevotella_9*, we identified *Prevotellaceae_UCG-003* and *Prevotellaceae_NK3B31* in the ATTR group by DEseq analysis. Also, in the HFrEF group, we found elevated levels of *Prevotella_7*. Members of this family have been reported to increase LPS levels in fatty liver disease [37].

In this study, one of our objectives was to investigate the relationship between gut microbiota diversity and inflammatory markers. We observed that ATTR patients, followed by HFrEF patients, showed a significant increase in the inflammatory profile, suggesting a potential association between disease severity and progression, and therefore a poorer prognosis in both ATTR and HFrEF patients. A recent study demonstrated that inflammation correlated significantly with increased mortality based on biopsy data from ATTR patients [38]. Hahn V et al. conducted a prospective study, using endomyocardial biopsies from HFrEF patients to identify histopathological phenotypes and their association with clinical characteristics, observing that there was evidence of a significant increase in inflammation in these patients [39].

The systemic inflammation present in ATTR patients could be associated with a significant increase in lipopolysaccharides (LPS). This increase in LPS levels could generate endotoxins from Gram-negative bacteria, which travel through the bloodstream, triggering low-grade endotoxemia, generating a systemic inflammatory response in the body and intestinal barrier dysfunction. This promotes a pro-inflammatory state due to the LPS translocation into the circulation [40].

Low-grade endotoxemia has been described in patients at risk of cardiovascular disease, and although it is not reported in our patients, it has been associated with the occurrence of cardiovascular events. It is known that one of the mechanisms that favors the development of low-grade endotoxemia is intestinal dysbiosis and changes in intestinal permeability [41].

On the other hand, it was observed that GM metabolites, such as SCFAs, showed a tendency to decrease in patients with ATTR or HFrEF; these are mainly acetic, isobutyric, and valeric acids, which are the main products associated with intestinal health. These SCFAs provide beneficial characteristics to colon cells, strengthen the intestinal barrier, and have relevant anti-inflammatory effects, regulating the immune system. They play a fundamental role in health and disease, as they regulate intestinal homeostasis, and their decrease is associated with various effects that can be harmful [42]. Currently, it is known that their deficiency is implicated in the pathogenesis of various disorders [43].

It is considered that the reduction in SCFA production could worsen the inflammatory state of patients with some types of cardiovascular disease due to increased bacterial translocation, as well as the presence of bacterial products in the systemic bloodstream [18]. Various GM metabolites have been linked to chronic degenerative diseases such as atherosclerosis, hypertrophic cardiomyopathy (HCM), chronic kidney disease, obesity, and T2DM among many others, including cardiovascular diseases such as heart failure. These metabolites could also contribute to ATTR amyloidosis and associated conditions such as reduced HFrEF [44].

In contrast, LCFAs, which have complex and sometimes opposing effects on cardiovascular risk (CVR), are generally associated with increased LDL cholesterol levels, thereby elevating the risk of cardiovascular disease. Although LCFAs are recognized as the primary energy substrates for myocardial metabolism, their excess can lead to adverse effects such as lipotoxicity, contributing to cardiac dysfunction [45].

The findings observed in our study may suggest a direct relationship with increased levels of troponin and proBNP, which were the most elevated and statistically significant biomarkers in patients with cardiac amyloidosis.

Regarding the enzymes and metabolic pathways documented to be statistically significant in the ATTR, HFrEF, and healthy groups, Li et al. demonstrated that glycerol dehydrogenase enzyme activity participates in the glycolytic pathway needed for the generation and metabolism of pyruvate from glucose [46]; on the other hand, other authors reported the enzyme Shikimate dehydrogenase as key to the development of antibiotics against methicillin-resistant *Staphylococcus aureus* [47]; these enzymes were found to be up-regulated only in the HFrEF group. On the other hand, of the enzymes reported as up-regulated in the healthy group, we only found previous reports by Cui et al. where the bacterium *Odoribacter splanchnicus* is a producer of GDP L-fucose and they also observed the repair of intestinal damage to P-glycoprotein related to aging [48]. Among the previously reported metabolic pathways, Muduli and colleagues associated the lysine biosynthetic pathway with a broad spectrum for new antibiotic targets [49]; however, Chen et al. showed that *Methanobrevibacter smithii* participates in chorismate biosynthesis, and this bacterium is also a potential indicator of aging [50], reported as enriched in our HFrEF group.

Furthermore, the aspartate superpathway metabolic pathway in gut microbiota was shown to confer protection against the development of idiopathic pulmonary fibrosis and chronic obstructive pulmonary disease [51]. Another research group showed that the cholanic acid building blocks biosynthesis pathway was enriched in the low co-occurring group of rectal cancer (n = 22) [52]; Liu et al. also revealed that Octenyl succinic anhydride starch favored the selective enrichment of beneficial intestinal microorganisms, such as *Faecalibaculum rodentium* and *Bifidobacterium adolescentis*, associated with greater activity of microbial metabolic functions, including the metabolism of pyruvate, butanoate and propanoate, as well as favorable changes such as an increase in pyruvate and a decrease in lactic acid, as well as improvements in the serum lipid profile and in markers of liver damage [53]. We documented that these pathways were enriched in the healthy controls.

## 4. Materials and Methods

### 4.1. Study Design

A cross-sectional, comparative, and analytical study was conducted after the endorsement and approvement of the Ethics and Research Committee of the National Institute of Cardiology “Ignacio Chávez” (INC) (protocol number 24-1447, reference INCAR-CBS-032). Furthermore, the study was conducted in accordance with the Declaration of Helsinki of the World Medical Association. Each enrolled patient provided written informed consent.

### 4.2. Study Cohort

**Figure 9:**
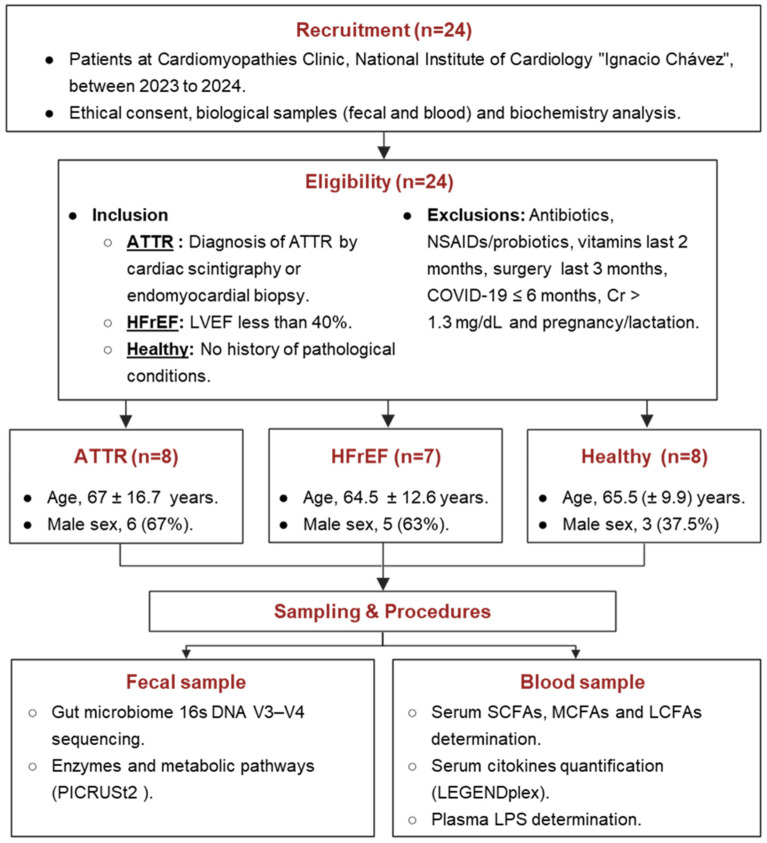
Flow chart. ATTR: amyloidosis TTR, HFrEF: heart failure with reduced ejection fraction, LCFAs: long-chain fatty acids, LVEF: left ventricular ejection fraction, MCFAs: medium-chain fatty acids, NSAIDs: non-steroidal anti-inflammatory drugs, SCFAs: short-chain fatty acids; PICRUSt2 Phylogenetics investigation of communities by reconstruction of unobserved states.

The inclusion criteria considered patients of both sexes, over 40 years of age, with a confirmed diagnosis of transthyretin amyloidosis by cardiac scintigraphy or endomyocardial biopsy. In addition, patients diagnosed with heart failure with reduced ejection fraction (HFrEF), as well as healthy controls from previously reported studies, were included. These control subjects were recruited during the same time period and were matched to the age group of ATTR patients. (Figure 9).

We excluded patients with light chain amyloidosis, other types of amyloidosis, and those with a history of current alcohol, tobacco or any other drug use, antibiotic and NSAIDs (non-steroidal anti-inflammatory drugs) use in the 2 months prior to the study, and a history of any type of surgery in the last 3 months. Additionally, participants were required to have no active infection at the time of sample collection.

### 4.3. Biological Samples

Whole blood was obtained by venipuncture from each patient. The samples were analyzed following the methodology established by the Central Laboratory of the National Institute of Cardiology “Ignacio Chávez” to obtain the biochemical profile. Subsequently, peripheral blood samples were collected from patients using polyethylene terephthalate tubes (BD Vacutainer, Franklin Lakes, USA) containing EDTA for plasma and polymer gel for serum. Plasma and serum were separated by centrifugation and immediately stored at −80 °C for future analysis.

Fecal samples were collected in a sterile container and kept refrigerated until delivery for a maximum period of eight hours. These samples were then immediately stored at −80 °C to preserve their integrity for subsequent analysis.

### 4.4. Genomic DNA Extraction and 16S RNA Sequencing

DNA extraction from fecal samples was conducted using the QIAamp PowerFecal Pro DNA Kit (QIAGEN, Hilden, Germany), also following the manufacturer’s protocol. The extracted DNA was sent to Novogene (Beijing, China) where 16S libraries were prepared and sequenced using the NovaSeq 6000 PE250 platform (Illumina, San Diego, CA, USA). In detail, the V3–V4 hypervariable region was amplified using the primer pair 341F (CCTAYGGGRBGCASCAG) and 806R (GGACTACNNGGGTATCTAAT).

### 4.5. Bioinformatics Analysis

Demultiplexed raw FastQ files were processed using QIIME2, v.2023.5. The DADA2 plugin was employed to merge paired-end FastQ files, denoise the data by removing chimeras and construct a table of operational taxonomic units (OTUs). The taxonomy assignment was conducted using the SILVA v.138 database at 99% identity, pre-trained for the V3–V4 region [14].

Alpha diversity was estimated using Observed OTUS, Shannon and Chao1 indexes while beta-diversity was assessed using unweighted unifrac and visualized through Principal Coordinates Analysis (PCoA) using phyloseq R package, v.1.54. Permutational Multivariate Analysis of Variance (PERMANOVA, 999 permutations) was applied to test for significant differences between groups using vegan R package, v.2.7. The core OTUs between groups were determined using the amp_venn function from the ampvis2 package, v.2.0, using the default parameters. Deseq2 analysis was performed using its default parameters using DEseq2 R Package, v.1.44.

### 4.6. Circulating Short-, Medium- and Long-Chain Fatty Acids Evaluation by GC-MS Analysis

FFAs, namely circulating SCFAs (acetic, propionic, butyric, isobutyric, 2-methylbutyric, isovaleric and valeric acids), MCFAs (hexanoic, isohexanoic, octanoic, decanoic and dodecanoic acids) and LCFAs (tetradecanoic, hexadecanoic and octadecanoic acids), were analyzed using anAgilent gas chromatography–mass spectrometry (GC–MS) system composed of a HP 5971 single quadrupole mass spectrometer, a HP 5890 gas chromatograph, and a HP 7673 autosampler (Santa Clara, CA, USA), following previously described protocols [54,55]. Briefly, prior to analysis each sample was thawed and FFAs were extracted as follows: 200 μL of serum sample was mixed with 10 μL of internal standard mixture, 100 μL of methyl tert-butyl ether, and 20 μL of 6 M HCl + 0.5 M NaCl solution in a 0.5 mL centrifuge tube. Tubes were vortexed for 2 min and centrifuged at 10,000 rpm for 5 min; the solvent layer was then transferred to a GC-MS vial fitted with a microvolume insert for analysis.

### 4.7. Cytokine Evaluation

Serum cytokines were quantified using the LEGENDplexTM Human Essential Immune Response Panel kit, 13plex (Biolegend, San Diego, CA, USA) that included the following cytokines: IL-2, IL-4, IL-6, IL-10, IL-17A, CXCL-8, CXCL-10, IL-1β, MCP-1, tumor necrosis factor (TNF), interferon-γ (IFN-γ), IL-12p70 and transforming growth factor-β1 (TGF-β1). Samples were evaluated using a FACSAria flow cytometer (Biosciences, San Jose, CA, USA) and raw data were analyzed with the LEGENDplex™ Data Analysis Software Suite. Each assay was performed in duplicate.

### 4.8. Lipopolysaccharide Test

LPS determination was performed using the Pierce Chromogenic QuantKit (Thermo Fisher Scientific, Rockford, IL, USA), following the manufacturer’s instructions. A 96-well endotoxin-free plate was used and equilibrated at 37 °C for 10 min. The endotoxin-free standard was then reconstituted. The standard curve was prepared using the stock solution at 1.0 EU/mL. Fifty milliliters of each sample was added to the wells containing the amebocyte lysate. The procedure was performed in the 96-well plate at 37 °C. The plate was incubated for 30 min. The chromogenic substrate was added, and the plate was incubated for 6 min. 25% acetic acid was added to each well to stop the reaction. The sample was then read at 405 nm.

### 4.9. Enzyme and Metabolic Pathway Predictions

We used PICRUSt2 (Phylogenetic Investigation of Communities by Reconstruction of Unobserved States) v2.4.1 [56] to predict the metabolic function of the genomes from the 16S rRNA gene dataset, with the KEGG and MetaCyc classification databases. The pipeline began by exporting the representative sequences to FASTA format and the abundance table to BIOM format from QIIME2. The unified picrust2_pipeline.py was then executed to perform sequence placement in a reference phylogenetic tree and initial predictions. Next, place_seqs.py placed the sequences into the reference tree using the SEPP method to generate a Newick format file. Using hsp.py, two types of hidden states were predicted: first, 16S marker gene copy numbers along with the NSTI index (measuring proximity to reference sequences) using the -n flag for normalization, followed by EC gene families using Maximum Likelihood. Subsequently, metagenome_pipeline.py combined the abundance table with marker copy and EC predictions to generate metagenome abundances using the --strat_out flag for stratified outputs, while convert_table.py transformed the contribution file to legacy format. For metabolic pathway inference, pathway_pipeline.py converted EC number predictions into pathways using the MetaCyc database with the MINPATH method. Finally, add_descriptions.py added functional descriptions to both EC numbers (from KEGG) and MetaCyc pathways, generating enriched files with names and descriptions to facilitate a biological interpretation of the results. For statistical analysis of enzymes and metabolic pathways, we used the pred_metagenome_unstrat_descrip.tsv and path_abun_unstrat_descrip.tsv files for analyses of enzymes and metabolic pathways respectively in RStudio (version 4.4.1). Both datasets were analyzed using an identical processing pipeline including normalization to relative proportions per sample, assignment to healthy, HFrEF, and ATTR groups with consistent factor levels, and application of uniform statistical parameters (α = 0.05, and bootstrap resampling with R = 2000). The statistical framework implemented a three-tier testing approach for each dataset for pairwise comparisons and reference-based testing using Welch’s *t*-test comparing healthy against the ATTR group and then the HFrEF group. Statistical results per dataset included means, standard deviation, and Welch’s *p*-values, and z-scores from healthy-referenced comparisons were used to obtain the top 20 significant enzymes and metabolic pathways in bar plots and heatmaps [56].

### 4.10. Statistical Analysis

Data was analyzed using the Statistical Package for the Social Sciences (SPSS, version 15.0) and GraphPad Prism 8. The Shapiro–Wilk test was applied to assess the normality of all quantitative variables. Student’s *t*-test for independent samples was used to compare mean differences. For non-parametric data, the Mann–Whitney U test was applied. The chi-square test was used for nominal variables, and the Kruskal–Wallis test was used for multiple quantitative variables. Graphical representations were generated using R software [v.4.1.2]. All analyses were two-tailed, and a *p* value ≤ 0.05 was considered statistically significant.

## 5. Conclusions

We present a comparative GM analysis in patients with ATTR amyloidosis or HFrEF. We observed the presence of members of *Prevotellaceae* as *Prevotella_9* and *Prevotellaceae_UCG-003* only in the ATTR group indicating that ATTR patients have an altered gut microbiota. Although HFrEF patients showed a significant increase in the pro-inflammatory profile, ATTR patients exhibited twice the expression of the same profile, suggesting a direct relationship with disease progression. The potential prediction of an adverse prognosis in patients with ATTR and patients with HFrEF based on changes in the GM diversity is suggested.

Even though the sample size can limit the findings of this study, we documented, for the first time, an altered gut microbiota in ATTR patients that could be associated with the presence of endotoxins, primarily caused by LPS, triggering low-grade endotoxemia and promoting a pro-inflammatory state due to its translocation into circulation.

Further studies with larger sample sizes, including additional markers of intestinal permeability such as ZO-1 protein and lipopolysaccharide-binding protein (LBP) are needed to confirm these findings, as well as measuring microbial metabolites to validate the metabolic pathways predicted from 16S data.

## Figures and Tables

**Table 1:** Clinical characteristics of patients with cardiac amyloidosis (ATTR) and heart failure with reduced ejection fraction (HFrEF).

Anthropometric and Laboratory Values	Healthy (*n = 8*)	HFrEF (*n = 7*)	ATTR (*n = 8*)	*p*
Age (years)	65.5 ± 9.9	64.5 ± 12.6	67 ± 16.7	0.929
Sex (male, %)	3 (37.5)	5 (63)	6 (67)	0.858
Body mass index (kg/m^2^)	22.8 (22–24)	24.5 (22–26)	26.4 (25–30) ^a,b^	0.002
Fasting glucose (mg/dL)	92 ± 9	94.6 ± 12)	101 ± 22	0.500
Total cholesterol (mg/dL)	205 ± 43	132 ± 33	155 (102–170)	0.9233
LDL cholesterol (mg/dL)	137 ± 36	80.2 ± 33 ^a^	89 ± 38 ^a^	0.009
HDL cholesterol (mg/mL)	50.6 (40–70)	40.7 (34–45)	39.8 (35–47)	0.165
Hs-CRP (mg/L)	0.51 (0.38–3.6)	1.91 (1.0–17)	7.3 (6.1–17.2) ^a^	0.045
Leukocytes (109/L)	4.57 ± 1.4	6.16 ± 0.76	6.95 ± 3.2	0.061
Serum albumin (g/dL)	4.47 ± 0.3	4.37 ± 0.5	4.0 ± 0.47	0.077
Troponin (ng/L)	10.8 (8–74)	26.0 (18–47)	48.8 (44–52)	0.127
ProBNP (pg/mL)	8.9 (8–84)	709 (491–7390) ^a^	4344 (425–11,829) ^a^	0.005
**Comorbidities**				
Obesity (*n*, %)	(0)	0 (0)	2 (22)	0.145
Current smoking (*n*, %)	0 (0)	2 (25)	0 (0)	0.099
Previous smoking (*n*, %)	1 (12.5)	0 (0)	3 (33)	0.165
Alcohol consumption (*n*, %)	0 (0)	1(13)	3 (33)	0.165
Dyslipidemia (*n*, %)	4 (50)	6 (75)	5 (56)	0.561
Hypertension (*n*, %)	0 (0)	8 (100) ^a^	6 (67) ^a^	<0.001
Type 2 diabetes (*n*, %)	0 (0)	7 (88) ^a^	5 (56) ^a^	0.002
Myocardial infarction (*n*, %)	NA	4 (50)	0 (0)	0.015
HFrEF (*n*, %)	NA	8 (100)	1 (11)	<0.001
**Pharmacological treatment**				
Aspirin use (*n*, %)	NA	6 (75)	1 (11)	0.008
Antihypertensive use (*n*, %)	NA	8 (100)	4 (44)	0.012
Hypoglycemic drugs (*n*, %)	NA	7 (88)	5 (56)	0.149
Statin use (*n*, %)	NA	6 (75)	1 (11)	0.008

Data are expressed as median (interquartile range) or number of subjects (%). *p*-values were calculated using the Kruskal–Wallis test for medians and the chi-square test for frequencies. ^a^ *p* < 0.005 vs healthy group and ^b^ *p* < 0.005 vs. HFrEF group. NA: not applied. HFrEF: heart failure with reduced ejection fraction; LDL: low-density lipoprotein; HDL: high-density lipoprotein; Hs-CRP: high-sensitivity C-reactive protein; proBNP: pro-brain natriuretic peptide.

**Table 2:** Functions of metabolic pathways statistically enriched among groups.

Pathway	Enriched	Function
Calvin–Benson–Bassham cycle	HFrEF	Carbon fixation pathway present in autotrophic organisms that converts CO_2_ into organic compounds (triose phosphates) using ATP and NADPH.
L-lysine biosynthesis I	HFrEF	Bacterial pathway for the synthesis of L-lysine via the diaminopimelate (DAP) pathway, essential for protein synthesis and cell wall formation.
Chorismate biosynthesis I	HFrEF	Produces chorismate through the shikimate pathway, a key precursor for aromatic amino acids, folates, ubiquinones, and other secondary metabolites.
Coenzyme A biosynthesis I	HFrEF	Pathway responsible for the synthesis of coenzyme A (CoA), a central metabolic cofactor involved in fatty acid metabolism.
Fucose degradation	HFrEF	Catabolic pathway that enables the utilization of L-fucose as a carbon and energy source, commonly associated with gut bacteria that metabolize host-derived glycans.
Glycogen biosynthesis I (from ADP-D-Glucose)	HFrEF	Pathway involved in the synthesis of glycogen, a polysaccharide used for intracellular carbon and energy storage in bacteria.
Glycogen degradation I (bacterial)	HFrEF	Breakdown of glycogen into glucose-1-phosphate or glucose, allowing mobilization of stored energy during nutrient limitation.
Superpathway of aromatic amino acid biosynthesis	HFrEF	Integrated pathway leading to the production of phenylalanine, tyrosine, and tryptophan from chorismate, linking central carbon metabolism to protein and secondary metabolite synthesis.
Superpathway of branched amino acid biosynthesis	HFrEF	Combined biosynthetic route for valine, leucine, and isoleucine, amino acids critical for protein synthesis and metabolic regulation.
Biotin biosynthesis I	Healthy	Pathway for the synthesis of biotin (vitamin B7), an essential cofactor for carboxylation reactions in fatty acid and amino acid metabolism.
Colanic acid building blocks biosynthesis	Healthy	Produces nucleotide–sugar precursors required for colanic acid synthesis, an extracellular polysaccharide involved in biofilm formation and bacterial stress protection.
Gluconeogenesis I	Healthy	Metabolic pathway that generates glucose from non-carbohydrate precursors such as pyruvate, lactate, or amino acids, maintaining carbon balance under low-glucose conditions.
Pyruvate fermentation to butanoate	Healthy	Anaerobic fermentation pathway converting pyruvate into butyrate, a short-chain fatty acid with key roles in host gut health and energy metabolism.
Superpathway of fatty acid biosynthesis initiation (*E. coli*)	Healthy	Describes the initial steps of bacterial fatty acid biosynthesis, including acetyl-CoA and malonyl-ACP formation, essential for membrane lipid production.

Data obtained from Metacyc database (https://metacyc.org/).

## Data Availability

Sequences of gut microbiota of ATTR and HFrEF patients are available at https://www.ncbi.nlm.nih.gov/sra/PRJNA1420466 (accessed on 16 April 2026). Sequences of a previous report were used as healthy patients: SRR33619669, SRR33619664, SRR33619673, SRR33619670, SRR33619666 and SRR33619667 from https://www.ncbi.nlm.nih.gov/sra/PRJNA1259703 (accessed on 16 April 2026).; SRR33973104 and SRR33973128 from https://www.ncbi.nlm.nih.gov/bioproject/PRJNA1276283 (accessed on 16 April 2026).

## Supplementary Materials

The following supporting information can be downloaded at https://www.mdpi.com/article/10.3390/ijms27093763/s1.

## References

[1] Garcia-Pavia P., Rapezzi C., Adler Y., Arad M., Basso C., Brucato A., Burazor I., Caforio A.L.P., Damy T., Eriksson U. (2021). Diagnosis and treatment of cardiac amyloidosis: A position statement of the ESC Working Group on Myocardial and Pericardial Diseases. Eur. Heart J..

[2] Rubin J., Maurer M.S. (2020). Cardiac Amyloidosis: Overlooked, Underappreciated, and Treatable. Annu. Rev. Med..

[3] Kittleson M.M., Maurer M.S., Ambardekar A.V., Bullock-Palmer R.P., Chang P.P., Eisen H.J., Nair A.P., Nativi-Nicolau J., Ruberg F.L., American Heart Association (2020). Cardiac Amyloidosis: Evolving Diagnosis and Management: A Scientific Statement From the American Heart Association. Circulation.

[4] Gertz M.A., Dispenzieri A., Sher T. (2015). Pathophysiology and treatment of cardiac amyloidosis. Nat. Rev. Cardiol..

[5] Siddiqi O.K., Ruberg F.L. (2018). Cardiac amyloidosis: An update on pathophysiology, diagnosis, and treatment. Trends Cardiovasc. Med..

[6] Gevaert A.B., Kataria R., Zannad F., Sauer A.J., Damman K., Sharma K., Shah S.J., Van Spall H.G.C. (2022). Heart failure with preserved ejection fraction: Recent concepts in diagnosis, mechanisms and management. Heart.

[7] Salzillo C., Franco R., Ronchi A., Quaranta A., Marzullo A. (2024). Cardiac Amyloidosis: State-of-the-Art Review in Molecular Pathology. Curr. Issues Mol. Biol..

[8] Xin Y., Hu W., Chen X., Hu J., Sun Y., Zhao Y. (2019). Prognostic impact of light-chain and transthyretin-related categories in cardiac amyloidosis: A systematic review and meta-analysis. Hell. J. Cardiol..

[9] Guijarro D., Eicher J.C., Bezard M., Piriou N., Sauer F., Roubille F., Costa J., Reant P., Donal E., Bauer F. (2025). Characteristics and Prognosis of Wild-Type Transthyretin Amyloid Cardiomyopathy Patients Diagnosed Before 65 Years Old. JACC Adv..

[10] Wixner J., Dispenzieri A., Amass L., Carlsson M., Riley S., Powers E., Kelly J.W., The THAOS investigators (2025). Survival in a Contemporary, Real-World Cohort of Patients with Mixed-Phenotype Transthyretin Amyloid Cardiomyopathy Treated with Tafamidis: An Analysis from THAOS. Cardiol. Ther..

[11] Nakahashi T., Arita T., Yamaji K., Inoue K., Yokota T., Hoshii Y., Fukunaga M., Nomura A., Watanabe H., Miura S. (2014). Impact of clinical and echocardiographic characteristics on occurrence of cardiac events in cardiac amyloidosis as proven by endomyocardial biopsy. Int. J. Cardiol..

[12] Cardoso I., Saraiva M.J. (2006). Doxycycline disrupts transthyretin amyloid: Evidence from studies in a FAP transgenic mice model. FASEB J..

[13] Obici L., Cortese A., Lozza A., Lucchetti J., Gobbi M., Palladini G., Perlini S., Saraiva M.J., Merlini G. (2012). Doxycycline plus tauroursodeoxycholic acid for transthyretin amyloidosis: A phase II study. Amyloid.

[14] Peterle D., Pontarollo G., Spada S., Brun P., Palazzi L., Sokolov A.V., Spolaore B., Polverino de Laureto P., Vasilyev V.B., Castagliuolo I. (2020). A serine protease secreted from Bacillus subtilis cleaves human plasma transthyretin to generate an amyloidogenic fragment. Commun. Biol..

[15] Heintz-Buschart A., Wilmes P. (2018). Human Gut Microbiome: Function Matters. Trends Microbiol..

[16] Marizzoni M., Cattaneo A., Mirabelli P., Festari C., Lopizzo N., Nicolosi V., Mombelli E., Mazzelli M., Luongo D., Naviglio D. (2020). Short-Chain Fatty Acids and Lipopolysaccharide as Mediators Between Gut Dysbiosis and Amyloid Pathology in Alzheimer’s Disease. J. Alzheimer’s Dis..

[17] Poll B.G., Cheema M.U., Pluznick J.L. (2020). Gut Microbial Metabolites and Blood Pressure Regulation: Focus on SCFAs and TMAO. Physiology.

[18] Tang W.H.W., Li D.Y., Hazen S.L. (2019). Dietary metabolism, the gut microbiome, and heart failure. Nat. Rev. Cardiol..

[19] Rissato J.H., de Melo Pereira N., Romero C.E., Del Cisne Jadan Luzuriaga G., Kerges Bueno B.V., Fonseca Cafezeiro C.R., de Alencar Neto A.C., Borges T.S., Freitas Carvalhal S., Ramires F.J.A. (2025). Different Gut Microbiome Profiles in Patients with Transthyretin Amyloidosis with and Without Cardiac Involvement. Int. J. Mol. Sci..

[20] Li H., Wang Z., He S., Zhao X., Wu Q., Sun Y., Fan Y., Hu X., Tian Z., Zhang S. (2025). Unraveling gut microbiome alterations and metabolic signatures in hereditary transthyretin amyloidosis. Microbiol. Spectr..

[21] Guo S., Al-Sadi R., Said H.M., Ma T.Y. (2013). Lipopolysaccharide causes an increase in intestinal tight junction permeability in vitro and in vivo by inducing enterocyte membrane expression and localization of TLR-4 and CD14. Am. J. Pathol..

[22] Hsu R.L., Lee K.T., Wang J.H., Lee L.Y., Chen R.P. (2009). Amyloid-degrading ability of nattokinase from *Bacillus subtilis* natto. J. Agric. Food Chem..

[23] Zhao L., Buxbaum J.N., Reixach N. (2013). Age-related oxidative modifications of transthyretin modulate its amyloidogenicity. Biochemistry.

[24] Suenaga G., Ikeda T., Komohara Y., Takamatsu K., Kakuma T., Tasaki M., Misumi Y., Ueda M., Ito T., Senju S. (2016). Involvement of Macrophages in the Pathogenesis of Familial Amyloid Polyneuropathy and Efficacy of Human iPS Cell-Derived Macrophages in Its Treatment. PLoS ONE.

[25] Luigetti M., Guglielmino V., Romano A., Sciarrone M.A., Vitali F., Sabino A., Gervasoni J., Primiano A., Santucci L., Moroni R. (2022). A Metabolic Signature of Hereditary Transthyretin Amyloidosis: A Pilot Study. Int. J. Mol. Sci..

[26] Ali A., Zhaliazka K., Dou T., Holman A.P., Kumar R., Kurouski D. (2023). Secondary structure and toxicity of transthyretin fibrils can be altered by unsaturated fatty acids. Int. J. Biol. Macromol..

[27] Liao G.Z., Liu H.H., He C.H., Feng J.Y., Zhuang X.F., Wang J.X., Zhou P., Huang Y., Zhou Q., Zhai M. (2024). Free fatty acids: Independent predictors of long-term adverse cardiovascular outcomes in heart failure patients. Lipids Health Dis..

[28] Di Mattia M., Sallese M., Lopetuso L.R. (2025). The interplay between gut microbiota and the unfolded protein response: Implications for intestinal homeostasis preservation and dysbiosis-related diseases. Microb. Pathog..

[29] Yamashita T. (2025). The role of gut microbiota in cardiovascular diseases and their potential as novel therapeutic targets. J. Cardiol..

[30] Constantino-Jonapa L.A., Aguilar-Villegas O.R., Hernandez-Ruiz P., Escalona-Montano A.R., Pallecchi M., Gonzalez-Pacheco H., Bartolucci G., Baldi S., Amezcua-Guerra L.M., Amedei A. (2025). The link between inflammatory/SCFA profiles and oral/gut microbiome: An observational study in patients with ST-segment elevation myocardial infarction. Curr. Res. Microb. Sci..

[31] Pecyna P., Bykowska-Derda A., Gabryel M., Mankowska-Wierzbicka D., Nowak-Malczewska D.M., Jaskiewicz-Rajewicz K., Jaworska M.M., Grzymislawski M., Dobrowolska A., Czlapka-Matyasik M. (2025). *Blautia* spp. in the gut microbiome: Relation to dietary choices and to the nutritional status of patients with irritable bowel syndrome. Nutrition.

[32] Hu J., Zhou C., Zhang L., Chen Y., Li J., Li J., Duan L. (2025). Exploring the role of gut microbiota in inflammatory bowel disease patients comorbid with non-alcoholic fatty liver disease. Gut Pathog..

[33] Xia Y., Xiao Y., Wang Z.H., Liu X., Alam A.M., Haran J.P., McCormick B.A., Shu X., Wang X., Ye K. (2023). Bacteroides Fragilis in the gut microbiomes of Alzheimer’s disease activates microglia and triggers pathogenesis in neuronal C/EBPbeta transgenic mice. Nat. Commun..

[34] Zafar H., Saier M.H. (2021). Gut Bacteroides species in health and disease. Gut Microbes.

[35] Hitch T.C.A., Bisdorf K., Afrizal A., Riedel T., Overmann J., Strowig T., Clavel T. (2022). A taxonomic note on the genus *Prevotella*: Description of four novel genera and emended description of the genera *Hallella* and *Xylanibacter*. Syst. Appl. Microbiol..

[36] Abdelsalam N.A., Hegazy S.M., Aziz R.K. (2023). The curious case of *Prevotella copri*. Gut Microbes.

[37] Yuan H., Wu X., Wang X., Zhou J.Y., Park S. (2024). Microbial Dysbiosis Linked to Metabolic Dysfunction-Associated Fatty Liver Disease in Asians: *Prevotella copri* Promotes Lipopolysaccharide Biosynthesis and Network Instability in the Prevotella Enterotype. Int. J. Mol. Sci..

[38] Siegismund C.S., Escher F., Lassner D., Kuhl U., Gross U., Fruhwald F., Wenzel P., Munzel T., Frey N., Linke R.P. (2018). Intramyocardial inflammation predicts adverse outcome in patients with cardiac AL amyloidosis. Eur. J. Heart Fail..

[39] Hahn V.S., Yanek L.R., Vaishnav J., Ying W., Vaidya D., Lee Y.Z.J., Riley S.J., Subramanya V., Brown E.E., Hopkins C.D. (2020). Endomyocardial Biopsy Characterization of Heart Failure With Preserved Ejection Fraction and Prevalence of Cardiac Amyloidosis. JACC Heart Fail..

[40] Delk S.C., Reddy S.T., Fogelman A.M. (2025). The intestine and cardiovascular disease. Curr. Opin. Lipidol..

[41] Violi F., Cammisotto V., Bartimoccia S., Pignatelli P., Carnevale R., Nocella C. (2023). Gut-derived low-grade endotoxaemia, atherothrombosis and cardiovascular disease. Nat. Rev. Cardiol..

[42] Hays K.E., Pfaffinger J.M., Ryznar R. (2024). The interplay between gut microbiota, short-chain fatty acids, and implications for host health and disease. Gut Microbes.

[43] Fusco W., Lorenzo M.B., Cintoni M., Porcari S., Rinninella E., Kaitsas F., Lener E., Mele M.C., Gasbarrini A., Collado M.C. (2023). Short-Chain Fatty-Acid-Producing Bacteria: Key Components of the Human Gut Microbiota. Nutrients.

[44] Hu T., Wu Q., Yao Q., Jiang K., Yu J., Tang Q. (2022). Short-chain fatty acid metabolism and multiple effects on cardiovascular diseases. Ageing Res. Rev..

[45] Golaszewska K., Harasim-Symbor E., Polak-Iwaniuk A., Chabowski A. (2019). Serum fatty acid binding proteins as a potential biomarker in atrial fibrillation. J. Physiol. Pharmacol..

[46] Li H.K., Zhi X., Vieira A., Whitwell H.J., Schricker A., Jauneikaite E., Li H., Yosef A., Andrew I., Game L. (2023). Characterization of emergent toxigenic M1_UK_ Streptococcus pyogenes and associated sublineages. Microb. Genom..

[47] Enriquez-Mendiola D., Tellez-Valencia A., Sierra-Campos E., Campos-Almazan M., Valdez-Solana M., Gomez Palacio-Gastelum M., Avitia-Dominguez C. (2019). Kinetic and molecular dynamic studies of inhibitors of shikimate dehydrogenase from methicillin-resistant *Staphylococcus aureus*. Chem. Biol. Drug Des..

[48] Cui C., Fang L., Li L., Lai X., Zhang R., Zhang Q., Miao R., Hu G., Zhang M., Sia J.E.V. (2025). *Odoribacter splanchnicus* rescues aging-related intestinal P-glycoprotein damage via GDP-L-fucose secretion. Nat. Commun..

[49] Muduli S., Karmakar S., Mishra S. (2023). The coordinated action of the enzymes in the L-lysine biosynthetic pathway and how to inhibit it for antibiotic targets. Biochim. Biophys. Acta Gen. Subj..

[50] Chen S., Zhang Z., Liu S., Chen T., Lu Z., Zhao W., Mou X., Liu S. (2024). Consistent signatures in the human gut microbiome of longevous populations. Gut Microbes.

[51] Chen L., Chen H., Li Q., Ma J., Feng Y., Zhang S., Han Y., Pan J., Zhang M., Sun K. (2025). The aspartate superpathway in gut microbiota-related metabolic pathways mediates immune cell protection against COPD and IPF: A Mendelian randomization analysis. Aging.

[52] Gonzalez-Mercado V.J., Lim J., Yu G., Penedo F., Pedro E., Bernabe R., Tirado-Gomez M., Aouizerat B. (2021). Co-Occurrence of Symptoms and Gut Microbiota Composition Before Neoadjuvant Chemotherapy and Radiation Therapy for Rectal Cancer: A Proof of Concept. Biol. Res. Nurs..

[53] Liu C., Liu T., Ma R., Pan X., Tian Y. (2025). Octenyl Succinic Anhydride Starch Alleviates Alcoholic Liver Disease by Modulating Gut Microbiota and Metabolism. Nutrients.

[54] Baldi S., Menicatti M., Nannini G., Niccolai E., Russo E., Ricci F., Pallecchi M., Romano F., Pedone M., Poli G. (2021). Free Fatty Acids Signature in Human Intestinal Disorders: Significant Association between Butyric Acid and Celiac Disease. Nutrients.

[55] Bartolucci G., Pallecchi M., Menicatti M., Moracci L., Pucciarelli S., Agostini M., Crotti S. (2022). A method for assessing plasma free fatty acids from C2 to C18 and its application for the early detection of colorectal cancer. J. Pharm. Biomed. Anal..

[56] Douglas G.M., Maffei V.J., Zaneveld J.R., Yurgel S.N., Brown J.R., Taylor C.M., Huttenhower C., Langille M.G.I. (2020). PICRUSt2 for prediction of metagenome functions. Nat. Biotechnol..

