# Supplementary material for: Gut–Heart Axis: Microbiome Involvement in Wild-Type Transthyretin Amyloidosis
Source: Int J Mol Sci. 2026 Apr 23;27(9):3763. doi: 10.3390/ijms27093763 (PMC13164311; doi:10.3390/ijms27093763)
Supplement: Supplementary file 1 [file ijms-27-03763-s001.zip › ijms-4220742-Supplementary Figure.pdf]

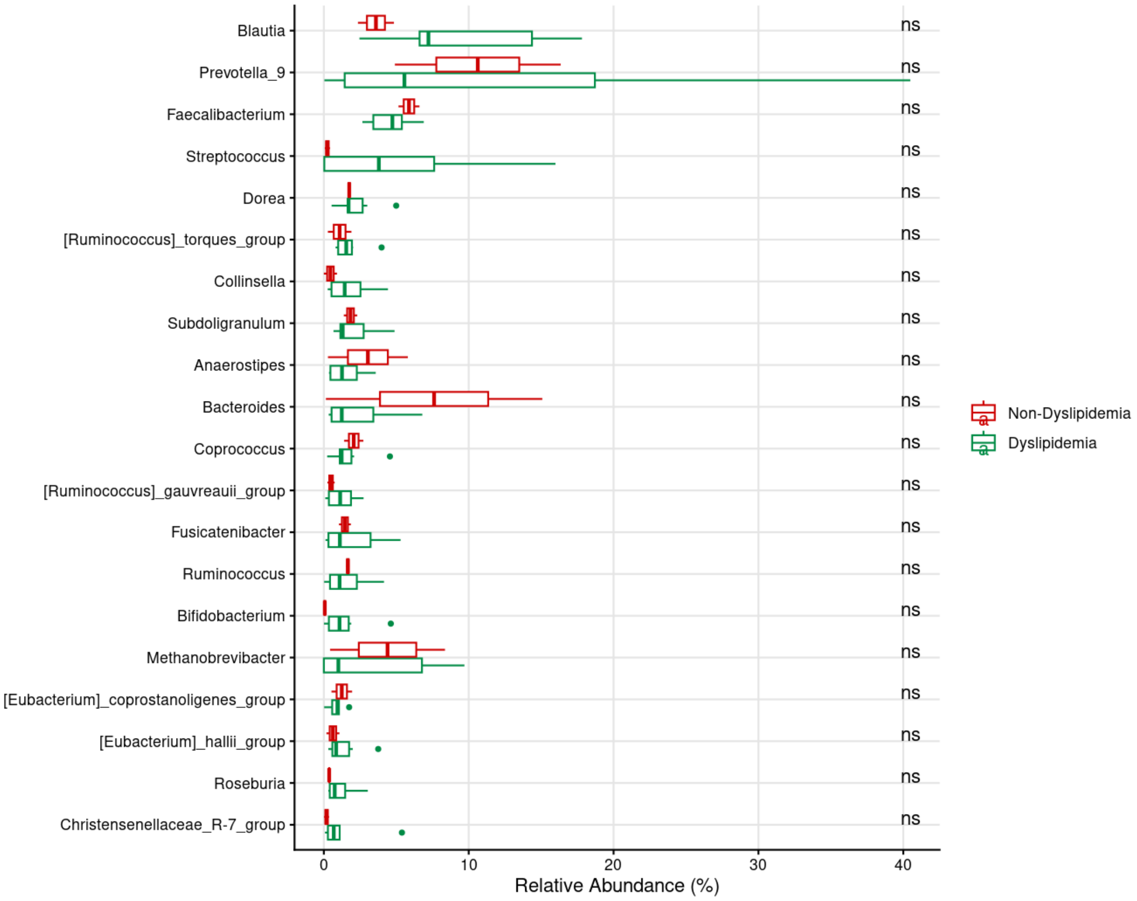

Supplementary Figure S1. Relative abundance of ATTR subjects grouped by dyslipidemia.

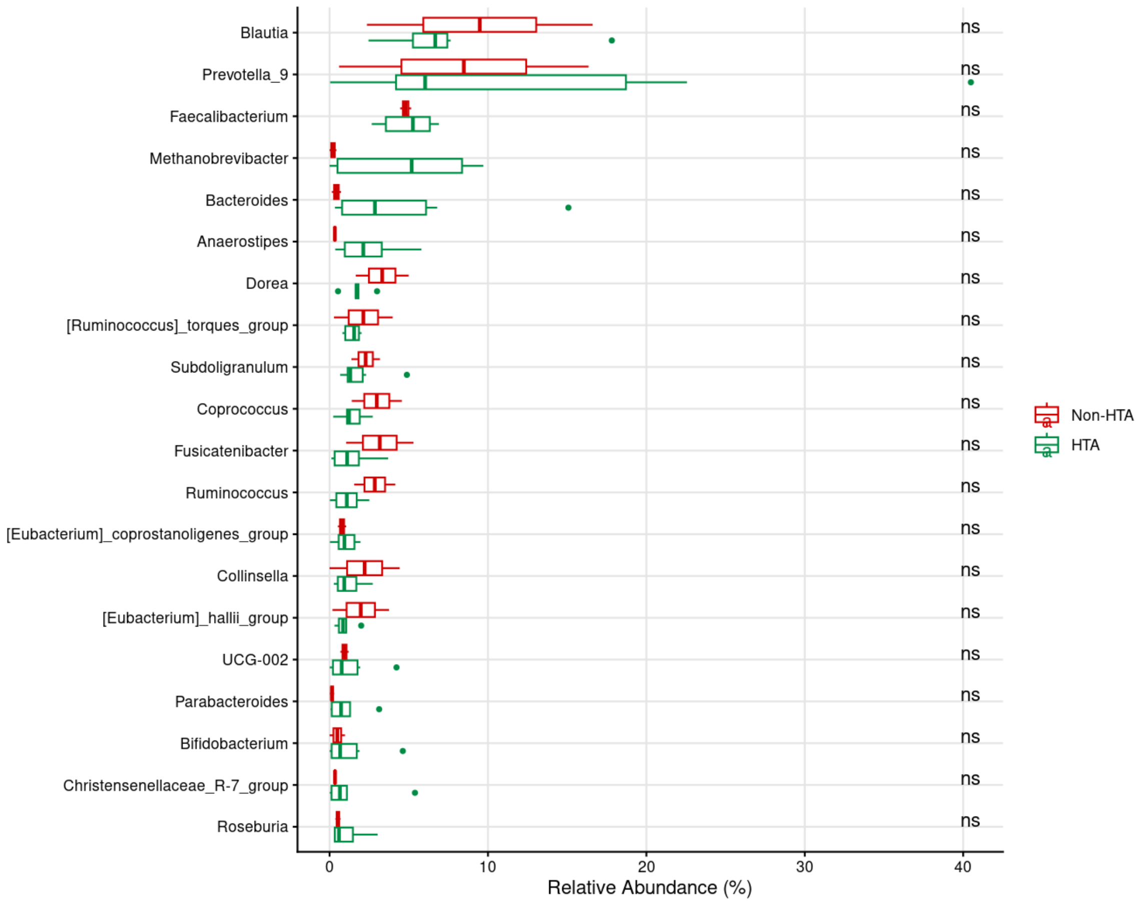

Supplementary Figure S2. Relative abundance of ATTR subjects grouped by hypertension (HTA).

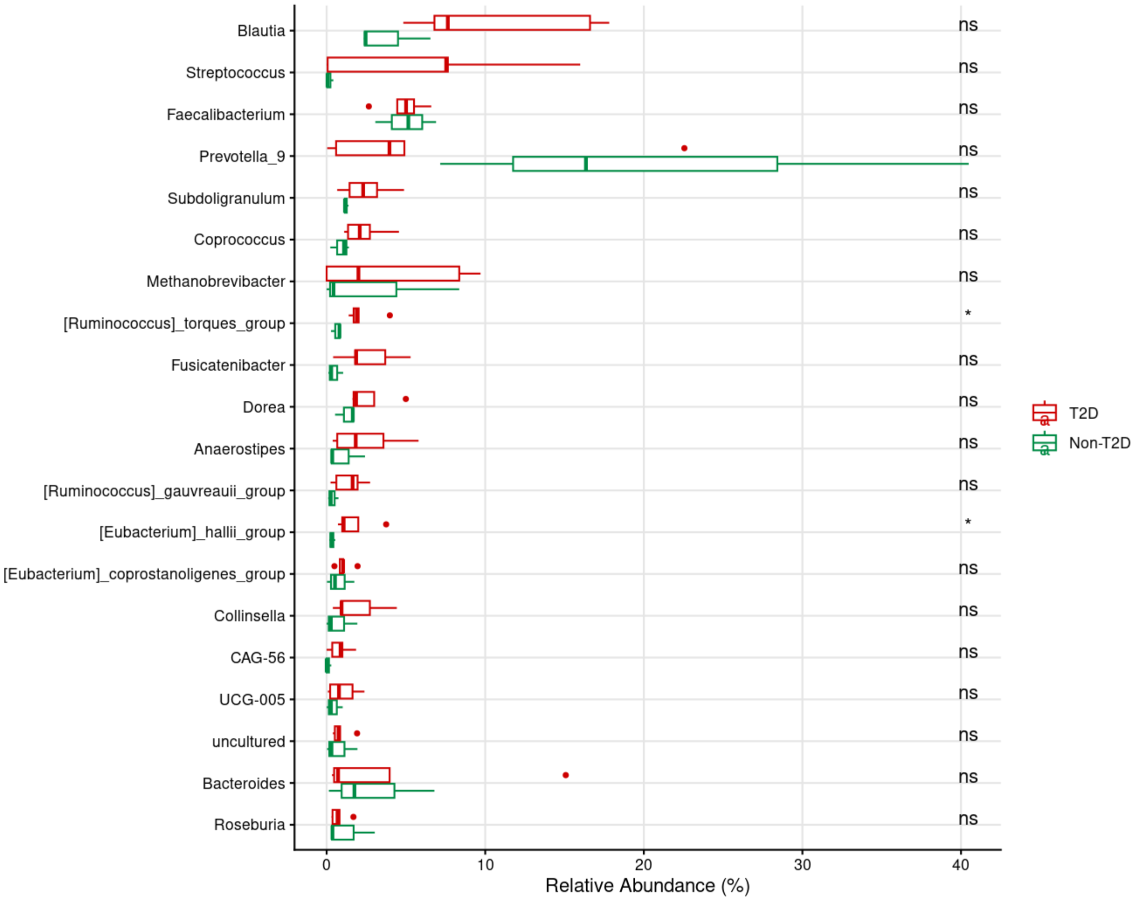

Supplementary Figure S3. Relative abundance of ATTR subjects grouped by type 2 diabetes (T2D).

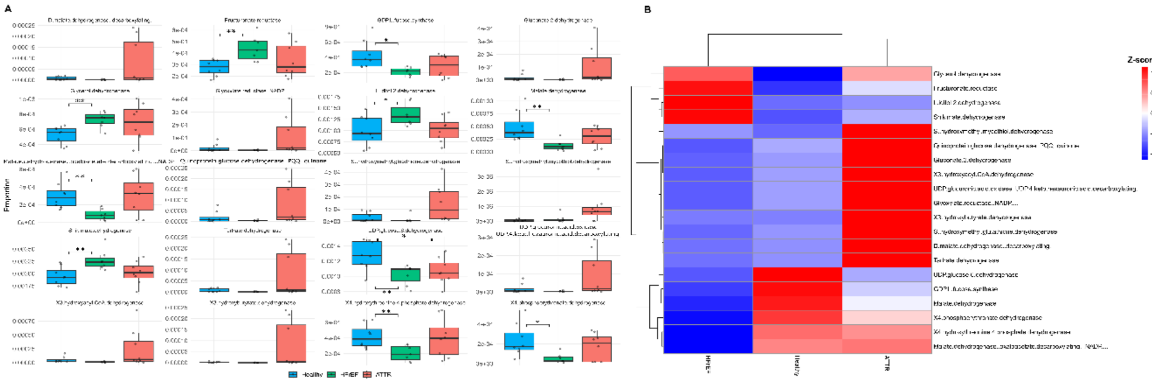

Supplementary Figure S4. Comparative analysis of enzymatic and metabolic pathway activities in patients with HFrEF and ATTR compared with healthy controls. **A**) Enzyme activity bar plot, **B**) Enzyme expression heatmap. **A;** Bar heights represent proportion of enzyme, colors denote distinct experimental groups; Healthy (Blue), HFrEF (Green) and ATTR (Red), **B;** Hierarchical clustering of enzyme expression profiles across groups and enzyme, rows correspond to individual enzymes and columns represent samples grouped by health condition, Z-score visualization of pathway activity shows a blue-to-red gradient that represents scores from -1 (downregulated) to +1 (upregulated). Asterisks denote statistical significance: *$p < 0.05$, **$p < 0.001$.

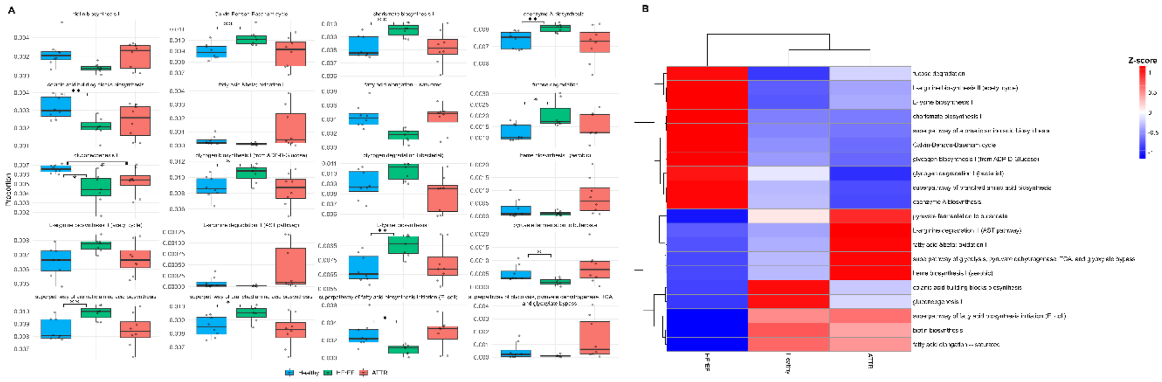

Supplementary Figure S5. Comparative analysis of enzymatic and metabolic pathway activities in patients with HFrEF and ATTR compared with healthy controls. **A**) Metabolic pathway activity bar plot, **B**) Metabolic pathway enrichment heatmap. **C**; Bar heights represent proportion of Metabolic pathway activity, colors denote distinct experimental groups; Healthy (Blue), HFrEF (Green) and ATTR (Red), **D**; Hierarchical clustering of metabolic pathways profiles across groups, rows correspond to individual pathways and columns represent samples grouped by health condition, Z-score visualization of pathway activity shows a blue-to-red gradient that represents scores from -1 (downregulated) to +1 (upregulated). Asterisks denote statistical significance: *$p < 0.05$, **$p < 0.001$.
